# Aortopulmonary fistula in a Warmblood mare associated with an aortic aneurysm and supravalvular aortic stenosis

**DOI:** 10.1111/jvim.15893

**Published:** 2020-09-14

**Authors:** Veronique Saey, Annelies Decloedt, Mario Van Poucke, Luc Peelman, Gunther van Loon, Katrien Vanderperren, Richard Ducatelle, Koen Chiers

**Affiliations:** ^1^ Department of Veterinary Pathology, Faculty of Veterinary Medicine Ghent University Merelbeke Belgium; ^2^ Department of Large Animal Internal Medicine, Faculty of Veterinary Medicine Ghent University Merelbeke Belgium; ^3^ Department of Nutrition, Genetics and Ethology, Faculty of Veterinary Medicine Ghent University Merelbeke Belgium; ^4^ Department of Veterinary Medical Imaging and Small Animal Orthopaedics, Faculty of Veterinary Medicine Ghent University Merelbeke Belgium

**Keywords:** aortic disease, elastin gene, equine, pseudoaneurysm

## Abstract

This case report describes the clinical presentation, the necropsy findings, and genetic results of a 13‐year‐old Warmblood mare presented with colic and a bilaterally loud, holosystolic murmur. Echocardiographic examination revealed the presence of a thoracic aortic aneurysm, an aortic pseudoaneurysm, a periaortic hematoma (circumferential cuffing by perivascular hemorrhage), and aortopulmonary fistulation. A supravalvular aortic stenosis (SVAS) was visible during echocardiography. Necropsy confirmed that the thoracic aortic aneurysm had ruptured and connected to the pseudoaneurysm, which fistulated into the pulmonary artery. Histologically, the aneurysm wall revealed chronic lesions such as fibrosis, mucin depositions, mineralizations, and elastin fragmentation. The mid abdominal aorta showed lesions suggestive of a systemic elastin arteriopathy. Molecular analysis, however, could not attribute this disease to a variant in the elastin gene, the most common causative gene for SVAS. To the authors' knowledge, this case report describes a case of aortopulmonary fistulation in a Warmblood horse associated with the presence of SVAS and an aortic aneurysm.

**Abbreviations**
ELN: elastin
SVAS: supravalvular aortic stenosis

## 1 INTRODUCTION

Three types of aortic stenosis exist in humans: subvalvular, valvular, and supravalvular.^1^

Supravalvular aortic stenosis (SVAS; Online database of Mendelian Inheritance in Man or OMIM #185500) is least common and is defined as a ridge of tissue distal to the aortic valve leaflets.^2^ Supravalvular aortic stenosis has an estimated incidence of 1:20 000 live births in humans and is often seen as part of the Williams‐Beuren syndrome (OMIM #194050), a neurodevelopmental genetic disorder.^3^, ^4^ Supravalvular aortic stenosis can also be inherited as a familial, nonsyndromic trait or can rarely manifest as an anomaly in human patients.^5^ SVAS is often associated with elastin (ELN; OMIM #130160) haploinsufficiency.^6^ In patients with an ELN arteriopathy, any artery can be affected. However, large systemic arteries, such as the thoracic aorta, are preferentially involved.^7^ Supravalvular aortic stenosis is also associated with a deranged vitamin D metabolism in the mother, the fetus, or both.^8^

Supravalvular aortic stenosis is rare in animals. A few cases have been mentioned in pigs but were not described in detail.^9^ Experimental SVAS has been induced in rabbit kittens after vitamin D administration to the doe, as seen in children born to mothers with hypervitaminosis D.^8^, ^10^ Supravalvular aortic stenosis is also rarely seen in dogs. An 18‐month‐old Schnauzer bitch was reported with SVAS.^11^ An affected Goldendoodle was treated successfully with high‐pressure balloon dilatation.^12^

Congenital cardiac defects are generally rare in horses.^13^ To the authors' knowledge, SVAS has been mentioned only once in a foal but no details were supplied.^14^

Although SVAS is described in several animal species, causal gene variants are only described in humans (>100) and only for approximately half of the patients.^15^

In all animal species, aortopulmonary fistulation is rare, except in Friesian horses, which seem to have a genetic predisposition for this disease in association with thoracic aortic rupture and pseudoaneurysm formation.^16^ Aortopulmonary fistulation associated with SVAS has not been reported in humans nor in animals.

## 2 CASE HISTORY

### 2.1 Clinical findings

A 13‐year‐old Warmblood mare was admitted to the Department of Large Animal Internal Medicine, Ghent University, with signs of acute colic. Three hours earlier, she had performed a normal jumping training session. The mare had been diagnosed with mild equine asthma 8 months before, which had been treated successfully. A left‐sided systolic murmur was noted when the illness anxiety disorder was diagnosed. However, no further cardiac examination was performed as this was thought to be a physiological murmur.

A decreased performance had been noticed by the owner for 3 months. At presentation, the heart rate was 65 beats per minute and regular. A holosystolic murmur, grade 5/6, was noticed both on the left and right sides. Gut sounds were reduced.

### 2.2 Echocardiography

**FIGURE 1:**
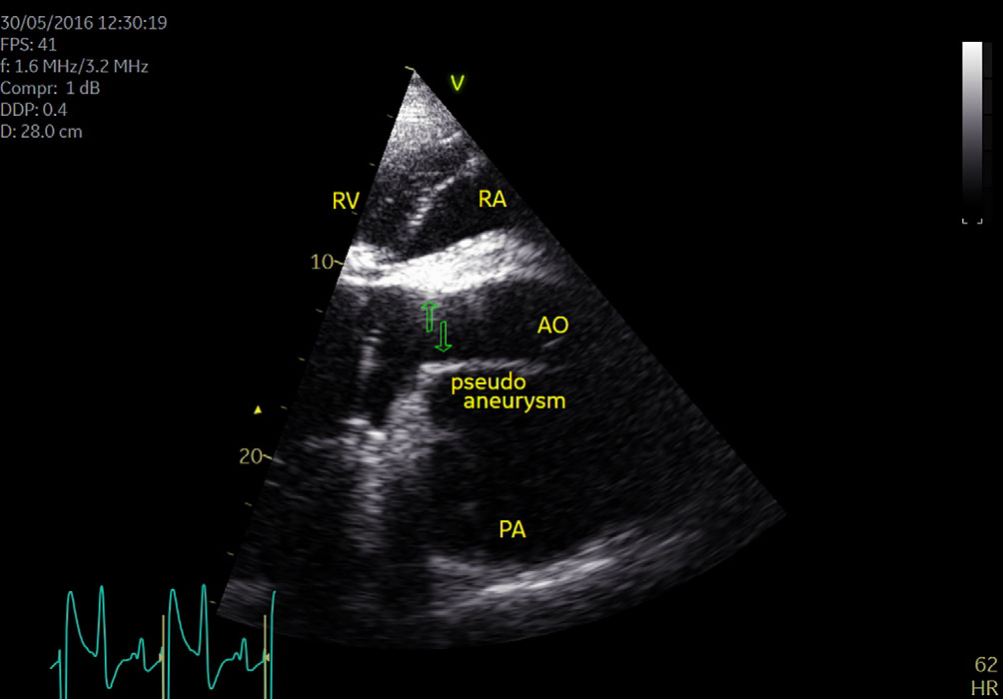
Two‐dimensional, right parasternal long axis, left ventricular outflow tract view of the aorta (AO), pulmonary artery (PA), and the pseudoaneurysm. Green arrows indicate the level of the aortic stenosis. RA, right atrium; RV, right ventricle

**FIGURE 2:**
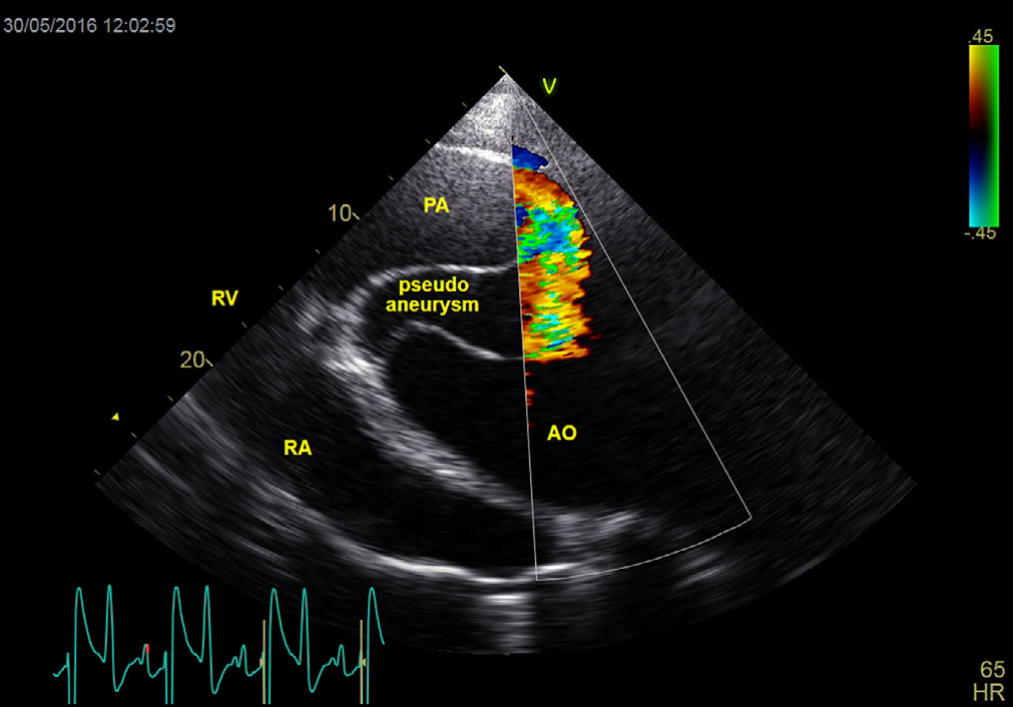
Two‐dimensional, left parasternal long‐axis view of the pulmonary artery (PA), aorta (AO), and the pseudoaneurysm with color Doppler flow. Turbulent blood flow from the aorta into the pulmonary artery is seen, indicating an aortopulmonary fistulation. RA, right atrium; RV, right ventricle

An aortic aneurysm, periaortic hematoma, and pseudoaneurysm associated with stenosis at the sinotubular junction (Figure 1), and an aortopulmonary fistulation were visualized on echocardiography (B mode). The systolic diameter of the aorta at the sinotubular junction was 4.1 cm, compared to the reference range of 5.6 to 7.6 cm. Mild left atrial enlargement and left ventricular hypertrophy were present. Doppler echocardiography showed a high‐velocity blood flow (3.90 m/s) in the proximal thoracic aorta, indicating aortic stenosis with a maximum pressure gradient of 61 mm Hg. There was turbulent blood flow in the aorta, pulmonary artery, and pseudoaneurysm (Figure 2). The horse was euthanized.

## 3 POSTMORTEM FINDINGS

### 3.1 Gross findings

**FIGURE 3:**
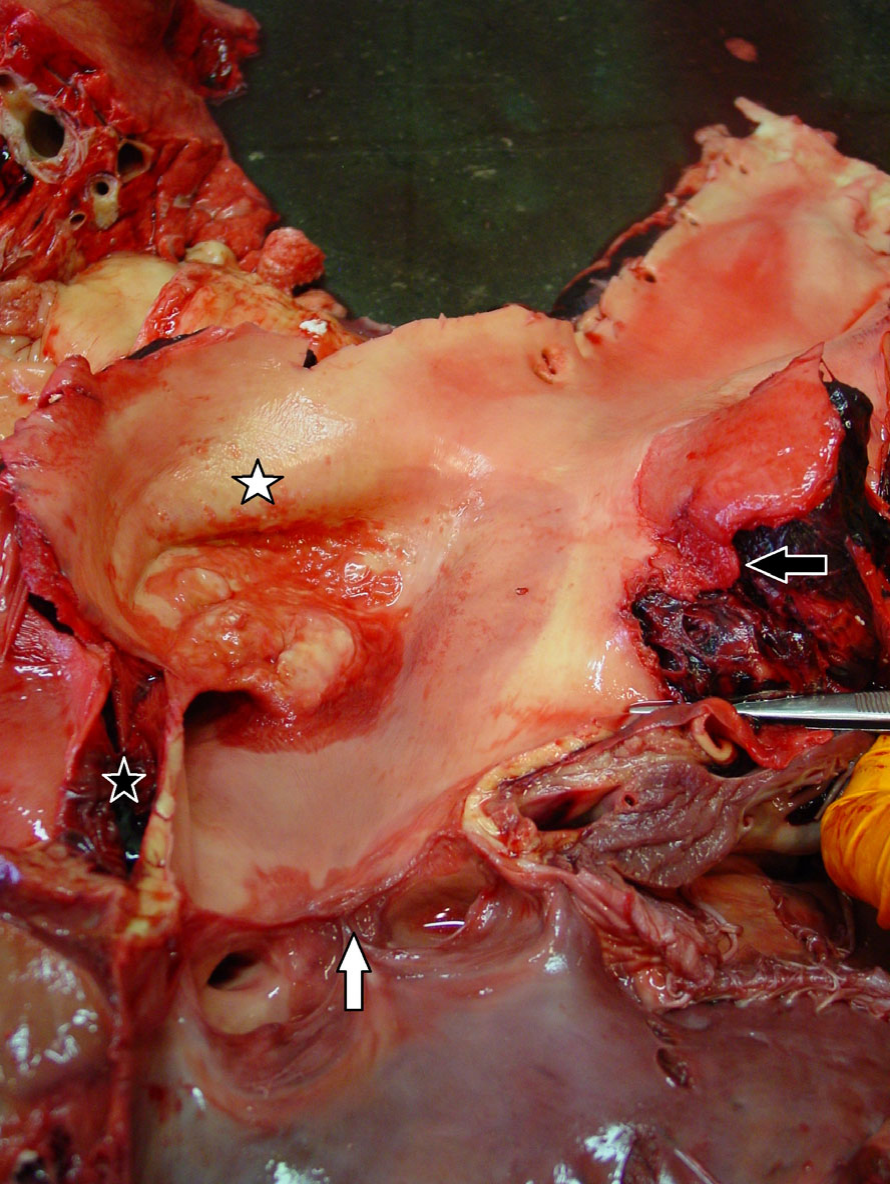
Thoracic aorta with circumferential stenosis (white arrow) immediately distal to the semilunar valves. An aortic aneurysm, delineated by a prominent irregular wall, is present (white star) distal to this stenotic area. The black arrow points to the aortic rupture. The black star represents the aortic dissection

A circumferential ridge immediately distal to the aortic valve leaflets was seen. Distal to this supravalvular stenosis, the thoracic aorta was markedly dilated over a length of 6 cm. The wall of this aneurysm was irregular with the presence of calcifications and jet lesions (Figure 3). The ventral side of the aortic aneurysm showed a 5 cm transverse rupture with irregularity of the intima. At this location, a dissection of the aortic wall over a length of 12 cm was noticed. The ruptured aortic aneurysm was connected to a pseudoaneurysm, which was located caudoventral to and partially surrounding the thoracic aorta and which fistulated into the pulmonary artery. A 4 cm transverse rupture of the pulmonary artery was located in the main vessel, just cranial to its bifurcation. Surrounding the first 50 cm of the thoracic aorta, a periaortic hematoma was present.

There was prominent left atrial dilatation and subendocardial fibrosis. The left ventricular wall showed concentric hypertrophy.

The lungs were moderately edematous with mild subpleural emphysema. The liver was diffusely congested.

### 3.2 Histopathology

Tissue samples of the aortic aneurysm, pseudoaneurysm, mid thoracic, and abdominal aorta were fixed in phosphate‐buffered formalin, embedded in paraffin wax and cut into 4‐μm‐thick sections. Sections were stained with hematoxylin and eosin, Alcian Blue (Sigma A4045‐25G, Zwijndrecht, the Netherlands, pH = 2.5), Van Gieson's stain (Klinipath 64 089, Duiven, the Netherlands), and Von Kossa stain. Aortic ELN was visualized with a monoclonal anti‐ELN antibody (clone BA‐4, Sigma). A standard avidin‐biotin complex method with diaminobenzidine as chromogen was used for visualization (Envision, Dako).

The aortic aneurysm wall showed a diffusely thickened and disorganized media with the presence of mucin depositions. The intima was irregular, wavy, and necrotic with depositions of fibrin and infiltrating neutrophils. The mid media displayed fragmentation and loss of ELN fibers with multifocal mineralization. Multiple deposits of aberrant collagen were present, mainly in the mid media (Figure 3).

At the level of the ruptured aortic aneurysm, there was extensive mid medial laminar necrosis. The adventitia was thickened because of fibrin depositions admixed with red blood cells, neutrophils, and proliferating fibroblasts. The intact intima and luminal half of the media were characterized by extensive hemorrhage and infiltration of neutrophils.

The wall of the pseudoaneurysm consisted of a thick layer of dense, fibrous tissue which had a diffusely necrotic aspect and was infiltrated by mainly neutrophils. In the surrounding fat tissue, fibrin depositions and large numbers of red blood cells were noticed, accompanied by proliferating fibroblasts.

**FIGURE 4:**
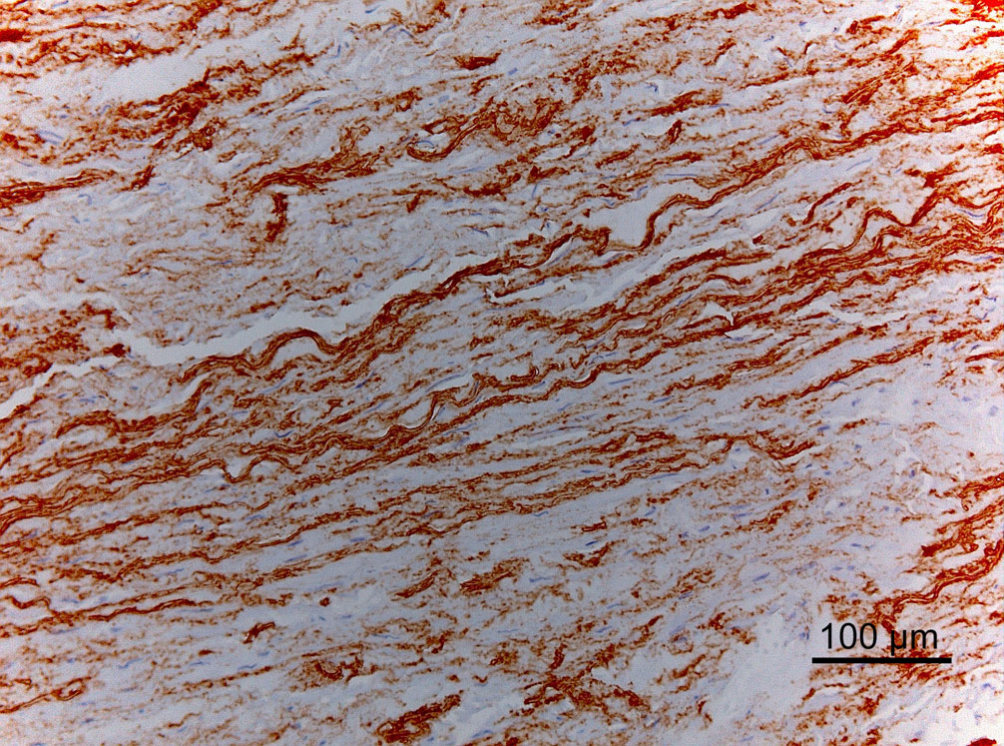
Abdominal aorta. Marked disorganization and fragmentation of elastin in the mid media. Immunohistochemical (IHC) staining for elastin, hematoxylin counterstain; ×200

**FIGURE 5:**
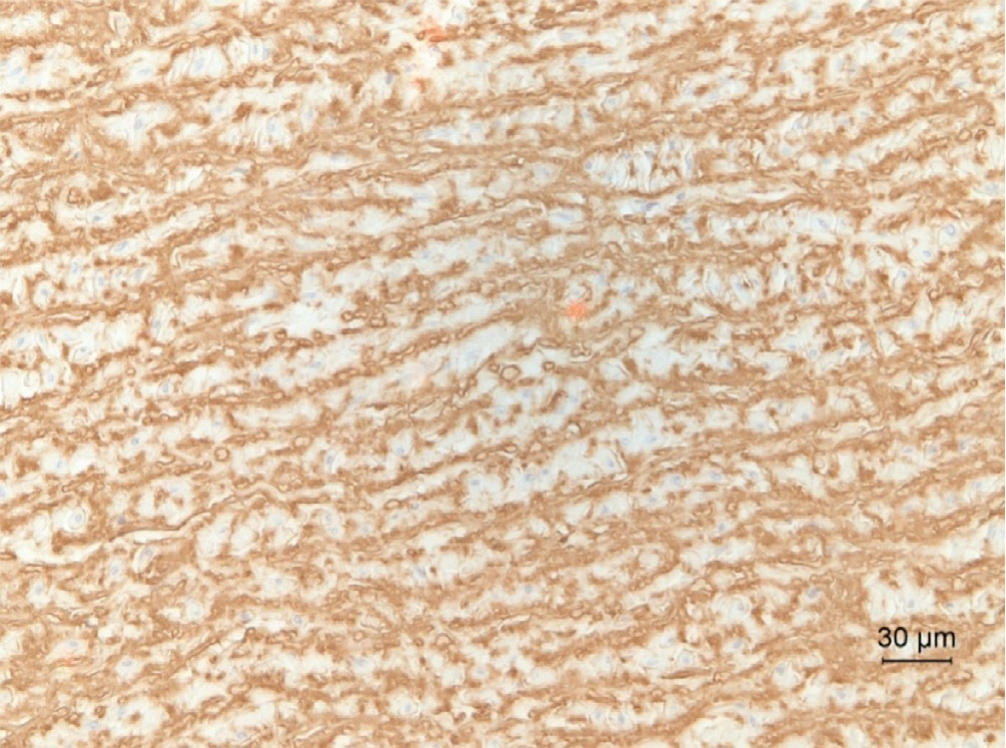
Lamellar organization of elastin in the mid media of the aorta. Control Warmblood horse. Immunohistochemical (IHC) staining for elastin, hematoxylin counterstain; ×200

The mid thoracic and mid abdominal aorta showed ELN fragmentation and cyst‐like lesions filled with basophilic ground substance throughout the media (cystic medial degeneration). This lesion was most prominent in the abdominal aorta (Figures 4, 5).

## 4 MOLECULAR ANALYSIS

Because all known causal variants for SVAS are so far exclusively found in ELN, a molecular genetic analysis of equine ELN (gene ID: 102150882; Genomic reference sequence: NC_009156.3 [11876538..11908454]) was performed (all experimental details are described in the Supplementary Information file). Unfortunately, there was no tissue available from related animals. As a reference, an aorta sample of an unrelated clinically healthy horse (that was euthanized because of case‐unrelated reasons) was used. Both aorta samples were taken immediately after euthanasia and stored at −20°C for approximately 22 months followed by DNA and RNA (converted to cDNA) extraction.

Three qPCR assays (covering the whole ELN gene and amplifying a part of intron 1, intron 15 and exon 36) indicated that there is no copy number variation, thus an ELN microdeletion was not the underlying genetic cause in this case. Next, the complete predicted equine ELN coding sequence was RT‐PCR amplified in 6 overlapping amplicons and Sanger sequenced. All predicted equine coding exons could be found in both the case and the control (Figure 5). Exon 7, known to be present in human transcripts but predicted not to be present in equine transcripts, was indeed not found. Besides the full‐length transcript, encoded by 35 exons and resulting in a coding sequence of 3253 bp (XM_023655460.1) and a protein of 728 amino acids (XP_023511228.1), also transcript variants missing exons 13, 33, and/or 35 were observed. Only 3 known variants were identified, 2 silent (rs394850339 and rs396110040) and 1 missense variant (rs394845532). The latter 1, NC_009156.3 (XM_023655460.1): c.61G>A (p.[V21I]), was not considered to be causal because the V_21_ is not conserved across mammals (I_21_ is also found in the porcine reference sequence) and the amino acid substitution is predicted to be neutral (75%) by PredictSNP.^17^

Because the RT‐PCR fragments from the case appeared to be weaker when analyzed on agarose gel than the ones from the control, RT‐qPCR was performed to check if there was a differential ELN mRNA expression between the case and the control, but there was none. In addition, the described negative cis‐acting regulatory element in intron 1 and the miRNA regulatory elements in the 3′‐UTR, were conserved and unchanged in the case.^18^, ^19^

No fragment could be RT‐PCR amplified in the case when forward primers were located upstream the ATG start‐codon, in contrary to the control. A region of 1000 bp upstream of the start‐codon could be amplified on genomic DNA from the case, but sequence analysis did not detect any variant. All described ELN promoter elements, such as the T‐Ex1 core, multiple transcription start sites, CAAT‐box, the lack of a TATA‐box, and AP‐2 and SP1 transcription factor binding sites were conserved in the case.^20^, ^21^ Adaptor‐free 5′‐RACE‐PCR was conducted to identify a potential alternative upstream exon in the case, but none was found.^22^ This confirms that there is no intron located 5′ to the ATG‐codon.^20^

## 5 DISCUSSION

This report describes a case of an aortopulmonary fistula associated with SVAS in an adult horse. The only case of SVAS in horses reported in literature until now involved a foal in which an increased left ventricular pressure was measured under anesthesia. No further details were supplied.^14^

To the authors' knowledge, aortopulmonary fistulation in association with SVAS has not been reported in animals nor in humans. In Friesian horses, aortic rupture and aortopulmonary fistulation is a well‐known disease that is typically associated with the formation of a pseudoaneurysm. The thoracic aorta in Friesians typically ruptures just proximal to the ligamentum arteriosum without abnormalities of the valves.^16^ A primary systemic collagen disorder has been suggested to be the cause of the aortic rupture in Friesians.^23^ In non‐Friesian horses, aortopulmonary fistulation is rare. A true aortic aneurysm fistulating into the left pulmonary artery was described in a 4‐year‐old hunter gelding.^24^ Another report reported a 3‐year‐old Dutch half‐bred horse suffering from a true thoracic aortic aneurysm.^25^ In both cases, an underlying cause of the aneurysm was not identified.

As SVAS is a congenital heart defect, undiagnosed cases in human adults are unusual.^26^ In cases of focal SVAS, as in our case, surgery yields good long‐term results in humans.^27^ Typical signs of SVAS in humans include angina, dyspnea, and syncope.^28^ The majority of human patients with nonsyndromic SVAS can remain asymptomatic until the age of 20 when sequelae develop.^29^ As SVAS is rare in animals, typical signs of this disease have not been described in detail. A 6‐month‐old Goldendoodle with SVAS remained asymptomatic and was evaluated for a heart murmur that was present since birth.^12^ An affected Schnauzer bitch was also asymptomatic until a sudden cough, dyspnea, and a change in temperament were noticed.^11^

Cardiovascular malformations associated with SVAS in humans include aortic coarctation and bicuspid aortic valve. The presence of these lesions can interfere with the diagnosis of SVAS.^26^, ^30^

In our case, the mare was asymptomatic until 13 years old when an aortopulmonary fistula had developed. Friesians affected by aortopulmonary fistulation may occasionally survive for weeks to months showing only vague signs.^16^ Surprisingly, considering the severeness of the lesions in this case, the mare had been ridden during jumping training a few hours before admission. Most likely, the aortic dissection and periaortic hematoma were more acute compared to the pseudoaneurysm, which presented an organized wall. Aortic dissection and periaortic hematoma are known to cause chest pain in human patients and were most likely the cause of the acute colic leading to hospital admission in the mare.^31^

Aortic aneurysm has only rarely been associated with SVAS in humans. A case of late aortic aneurysm formation in a human patient was reported following pericardial patch repair of SVAS.^32^ A 4‐year‐old boy who suffered from a familial form of SVAS developed several aortic aneurysms between his first and fourth year of life.^33^

As SVAS is so rare in animals, information regarding possible complications in animals is lacking. The cystic medial degeneration in both the thoracic and abdominal aorta in this case suggests the presence of an underlying, possibly inherited, connective tissue disorder, resulting in aortic weakness and aneurysm formation as seen in human Marfan syndrome.^34^ In humans, cystic medial degeneration has mainly been associated with aortic dissection.^35^ In the present case, SVAS could have contributed to the aortic aneurysm formation, as blood turbulence was seen at that level and jet lesions were found.

Information regarding related animals was not available. Molecular analysis demonstrated that no microdeletion, a structural variant, alternative splicing nor differential mRNA expression of ELN is the underlying genetic cause in this case. It remains, however, unclear why the 5′‐UTR appeared not to be present in the ELN transcripts of the case. Although it does not seem to have an impact at the mRNA level, it would be interesting to investigate if it would have an effect on protein level and thus cause this disease.

## CONFLICT OF INTEREST DECLARATION

Authors declare no conflict of interest.

## OFF‐LABEL ANTIMICROBIAL DECLARATION

Authors declare no off‐label use of antimicrobials.

## INSTITUTIONAL ANIMAL CARE AND USE COMMITTEE (IACUC) OR OTHER APPROVAL DECLARATION

Authors declare no IACUC or other approval was needed.

## HUMAN ETHICS APPROVAL DECLARATION

Authors declare human ethics approval was not needed for this study.

## Supporting information


**AppendixS1** Supporting information

**Video 1** Supplementary file: loop image, 2‐dimensional, right parasternal long axis, 4 chamber view. RV: right ventricle; RA: right atrium; LV: left ventricle; LA: left atrium.

**Video 2** Supplementary file: loop image, 2‐dimensional, left parasternal long axis view, with color Doppler flow.

**Video 3** Supplementary file: loop image, 2‐dimensional, right parasternal long axis, left ventricular outflow tract view with color Doppler flow. RV: right ventricle; RA: right atrium; AOV: aortic valves; AO: aorta.
