# Supplementary material for: Aortopulmonary fistula in a Warmblood mare associated with an aortic aneurysm and supravalvular aortic stenosis
Source: J Vet Intern Med. 2020 Sep 14;34(5):2152–7. doi: 10.1111/jvim.15893 (PMC7517844; doi:10.1111/jvim.15893)

Supplementary Information File. Description of the molecular genetic analysis of equine ELN (NCBI Gene ID: 102150882). A schematic overview showing the equine ELN gene structure, its full-length mRNA, amplified regions and found nucleotide variants is shown in Figure 3 at the end of this document.

1. DNA extraction and quality control

One hundred mg frozen aorta tissue was chopped in small pieces with a scalpel and digested overnight at 56°C in 500 µl digestion buffer (100 mM NaCl; 10 mM Tris-HCl, pH 8; 25 mM EDTA, pH 8; 0.5 % SDS; 200 µg proteinase K) while rotating. DNA was purified by phenol/chloroform extraction of the centrifugated supernatans, followed by an ethanol precipitation. DNA purity and concentration was evaluated and approved by Nanodrop analysis. The DNA integrity and amplificability was evaluated and approved with the UBC integrity assay (Van Poucke and Peelman, 2017; Figure 1.B). (q)PCR reactions were performed with 20 ng DNA.

2. RNA extraction, reverse transcription and quality control

One hundred mg frozen aorta tissue was chopped in small pieces with a scalpel and total RNA was isolated in a volume of 30 µl with the Aurum Total RNA Fatty and Fibrous Tissue Kit (Bio-Rad) according to the manufacturer's instructions, including an on-column DNase treatment. RNA purity and concentration was evaluated and approved by Nanodrop analysis. RNA integrity, evaluated by agarose gelelectrophoresis (Figure 1.A), showed that the RNA of the case was partly degraded, while the RNA of the control was only slightly degraded. Minus RT-PCR with the UBC integrity assay showed that there was a trace of degraded genomic DNA in the case, but not in the control (Figure 1.B). One µg of total RNA was converted to cDNA with ImProm-II Reverse Transcriptase (Promega) using random hexamers and oligo-dT, and diluted 10 times. cDNA integrity and amplificability was determined with the UBC integrity assay (Figure 1.B). Despite the partial RNA degradation, fragments of 821 bp could be amplified with cDNA as a template from both the case and the control. Primer design was performed in a way to minimize a potential effect of the RNA degradation (smaller amplicons) or DNA contamination (intron-spanning primers and e-PCR check). RT-(q)PCR was performed with 2 µl diluted cDNA (the equivalent of 10 ng converted RNA).

Figure 1.A. Agarosegel showing the 1 kb+-ladder (1) and 1 µg of RNA from the case (2) and the control (3). **B.** Agarosegel showing the 1 kb+-ladder (1) and the fragments generated with the UBC integrity assay on 20 ng RNA from the case (2) and the control (3), cDNA from the case (4) and the control (5), 20 ng gDNA from the case (6) and a no template control (7).

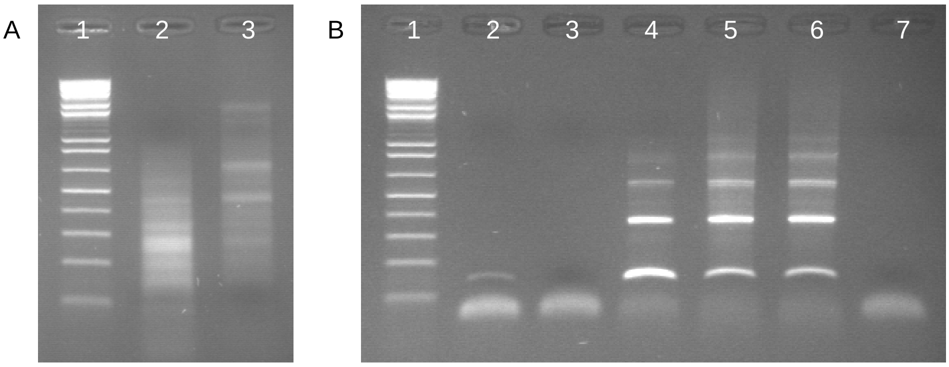

3. ELN exon description (Table 1)

Table 1. Location of the human and equine coding ELN exon sequences in their respective reference sequences. Exons were numbered as in Bashir *et al.* (1989).

EXON (CDS)	Human gDNA: NG_009261.1	Equine gDNA: NC_009156.3	Equine cDNA: XM_023655460.1
1	5092..5173	11876636..11876717	98..179
2	12268..12318	11882811..11882861	180..230
3	13459..13488	11883764..11883793	231..260
4	14611..14643	11884326..11884358	261..293
5	18120..18155	11887740..11887775	294..329

6	19518..19610	11888287..11888379	330..422
7	19888..19938	na	na
8	20022..20072	11888790..11888864	423..497
9	20783..20824	11889562..11889609	498..545
10	22126..22197	11890569..11890640	546..617
11	23092..23121	11890971..11891000	618..647
12	23600..23671	11891514..11891585	648..719
13	24579..24620	11892246..11892287	720..761
14	25046..25105	11892717..11892776	762..821
15	25407..25460	11893039..11893086	822..869
16	28654..28743	11894546..11894641	870..965
17	28828..28887	11894726..11894779	966..1019
18	30067..30213	11895377..11895550	1020..1193
19	31620..31673	11896553..11896606	1194..1247
20	33175..33339	11897197..11897295	1248..1346
21	33576..33617	11897527..11897568	1347..1388
22	34286..34372	11897961..11898047	1389..1475
23	34544..34600	11898900..11898959	1476..1535
24	36790..36951	11899970..11900041	1536..1607
25	37044..37088	11900125..11900184	1608..1667
26	37280..37405	11900760..11900864	1668..1772
27	38005..38043	11901550..11901588	1773..1811
28	40057..40128	11902979..11903050	1812..1883
29	40214..40273	11903135..11903182	1884..1931
30	40525..40599	11903439..11903528	1932..2021
31	42598..42636	11904225..11904263	2022..2060
32	42848..42901	11904474..11904527	2061..2114
33	43636..43680	11904910..11904954	2115..2159
34	na	11905357..11905386	2160..2189
35	na	11905962..11906012	2190..2240
36	45561..45604	11907442..11907485	2241..2284

4. ELN CNV analysis via qPCR

Three qPCR assays (targeting intron 1, intron 15 and exon 36 of equine ELN) were designed with Primer-BLAST (Ye *et al.*, 2012) to determine potential copy number variation (CNV; Table 2). The 3 ELN assays and 1 RPL32 assay (reference gene for normalization; Bogaert *et al.*, 2006), were performed in duplicate on 10 ng, 1 ng and 100 pg genomic DNA of the case and the control. All assays amplified the expected product without primer dimer formation, and showed a PCR efficiency of around 100 %, a correlation coefficient of around 1 and a dynamic range between Cq 23 and Cq 30. For all 4 assays and for both the case and the control all 6 measurements (3 dilution points in duplicate) were first averaged. The averaged Cq-value of every ELN assay was then normalized with the averaged Cq-value of the reference gene for both the case and the control. Finally, for all 3 ELN assays, the normalized Cq-values for both the case and the control were compared. The difference in Cq-value between the case and the control for all 3 ELN assays was lower than 0.1, proving that there was no ELN CNV between the case and the control.

Table 2. Details of the qPCR assays.

Primer name	Primer sequence (5'->3')	Amplicon position (NC_009156.3)	qPCR amplicon length (gDNA) Ta
ELN-1F<br>ELN-1R	AGCCGCAGTGAGCTGTGACCT<br>TGCAACCTTGTAATCAACTTAACCCTGACC	11877149 (intron 1)..<br>11877244 (intron 1)	96 bp<br>66°C
ELN-2F<br>ELN-2R	CCCAGAGGAGATTGTGCTGGTGAA<br>ATGGAGACAGTGCAGATGTGGCT	11894440 (intron 15)..<br>11894537 (intron 15)	98 bp<br>64°C
ELN-3F<br>ELN-3R	GGGAAAGCCTGTGGCCGGAAGA<br>GGGTTTACATTCTCCACCAAGCAGTAGCA	11907456 (exon 36, CDS)..<br>11907554 (exon 36, 3'-UTR)	99 bp<br>67°C

**qPCR mix** (Sopachem)	**qPCR cycling parameters** (CFX96 Real-Time PCR Detection System, Bio-Rad)
5 µl KAPA SYBR FAST Universal TDS<br>1 µl Primer mix (5 µM each primer)<br>2 µl H_2O<br>2 µl Template<br>10 µl Total volume	1. 95°C for 3:00<br>2. 95°C for 0:20<br>3. Ta°C for 0:40<br>+ Plate read<br>4. GOTO 2, 39 more times<br>5. Melt curve 72°C to 92, increment 0.5°C for 0:05 + Plate Read<br>END

5. ELN variant analysis via RT-PCR and Sanger sequencing

Multiple RT-PCR assays were designed with Primer-BLAST to amplify the complete equine ELN coding sequence with cDNA from the case and the control as a template (Table 3). The amplicons were analyzed on a 1 % agarosegel. The amplicons from the case were eluted and sequenced via Sanger sequencing (Table 4). The chromatograms were analyzed with UGENE (Okonechnikov *et al.*, 2012) and the sequences were compared with the equine transcript variant 1 mRNA sequence (XM-_023655460.1) using BLAST (Altschul *et al.*, 1990). Amplicons were generated in both the case and the control with all primer pairs, except for primers located upstream the start codon. Those could only generate an amplicon in the control, but not in the case. Amplicons from the control sample always appeared stronger on gel than from the case sample. Transcript variants missing exon 13, 33 and/or 35 were found in both case and control. Three SNVs were detected in the case sequence when compared to the mRNA reference sequence, but none of them were found to affect ELN function (Figure 3).

Table 3. Details of the RT-PCR assays. Some assays performed better by adding 1 µl GC-rich (Roche) to the RT-PCR mix at the expense of 1 µl H_2O.

Primer name	Primer sequence (5'->3')	Amplicon position (XM_023655460.1)	RT-PCR amplicon length (cDNA) Ta + RT-PCR mix info
ELN-4F<br>ELN-4R	GATGGCGGGTCTGACAGCTAC<br>ACCAGCCTTGGCAGCTTTATAGG	97 (exon 1, CDS)..<br>424 (exon 6)	328 bp<br>60°C
ELN-5F<br>ELN-5R	GGGACCTGGAGGCAAACCAGCC<br>GCTTCCCAGTGCTGTAAGGCAGTCC	265 (exon 4)..<br>752 (exon 13)	488 bp<br>65°C
ELN-6F<br>ELN-6R	CGCCGTAGGGAAGCCTGGGAAA<br>CAGGGACTCCAACTCCAGCAGCTC	469 (exon 8)..<br>1043 (exon 18)	575 bp<br>65°C + GC-rich
ELN-7F<br>ELN-7R	CCGGAGTTCTCCCGGGCGTT<br>GGCTGCTTTGGCGGCTGCTTTA	882 (exon 16)..<br>1240 (exon 19)	359 bp<br>65°C + GC-rich
ELN-8F<br>ELN-8R	GGAGTCCCAGGTGTTGTTGGACCAG<br>TGCGGCCAGGGGTCCAGGTA	1169 (exon 18)..<br>1792 (exon 27)	624 bp<br>65°C + GC-rich
ELN-9F<br>ELN-9R	TGGTGCTGCTGTTCCCGGAC<br>GAGGTCGTGAGTCAGGGGTCCT	1711 (exon 26)..<br>2313 (exon 36, 3'-UTR)	603 bp<br>65°C + GC-rich
ELN-10F<br>ELN-10R	CCTACGAGGCAGCAATTACGCTTTGGG<br>GCCCGCCAACACCTGCCTTG	26 (exon 1, 5'-UTR)..<br>305 (exon 5)	280 bp<br>65°C + GC-rich

ELN-11F<br>ELN-11R	TGGGGCATTTCTCCCCGA<br>CCAGCCTTGGCAGCTTTATAGG	79 (exon 1, 5'-UTR)..<br>423 (exon 6)	345 bp<br>64°C

**RT-PCR mix** (VWR International)	**RT-PCR cycling parameters** (S1000, Bio-Rad)
5.7 µl H_2O<br>1.0 µl 10x Key buffer<br>1.0 µl PCR primer mix (5 µM each primer)<br>0.2 µl dNTPs (10 mM each nucleotide)<br>0.1 µl TEMPase HS DNA Polymerase (5 U/µl)<br>2.0 µl Template<br>10.0 µl Total volume	14'30" - 95°C<br>00'30" - 95°C]<br>00'30" - Ta°C] x40<br>01'00" - 72°C]<br>04'00" - 72°C<br>HOLD - 15°C

Table 4. Details on the Sanger sequencing. Sequencing reactions were performed on amplicons eluted from the agarosegel using the GENECLEAN II kit (mpbio; according to the manufacturer's instructions) with a PCR primer and analyzed on an ABI 3730 XL sequencing machine at Eurofins Genomics (Germany).

**Sequencing Mix** (BigDye Terminator v3.1 Cycle Sequencing Kit, AB)	**Sequencing cycling parameters** (S1000, Bio-Rad)
3.0 µl H_2O<br>0.5 µl RR-mix<br>2.0 µl 5x sequencing buffer<br>1.0 µl GC-rich (Roche)<br>1.5 µl PCR primer (2 µM)<br>2.0 µl eluted PCR product<br>10.0 µl total volume	02'00" - 95°C<br>00'20" - 95°C]<br>00'15" - 60°C] x30<br>04'00" - 65°C]<br>HOLD - 04°C

6. Analysis of the equine ELN exon 1 area and potential regulatory elements on gDNA level via PCR and Sanger sequencing

Because the 5'-UTR of ELN could not be RT-PCR amplified with cDNA of the case as a template (in contrary to the control), the area around exon 1 was analyzed at gDNA level in the case (Table 5). The whole area could be PCR amplified and no nucleotide variants were found in the sequence when compared with the equine genomic reference sequence (NC_009156.3), including the T-EX1 core sequence (Pierce *et al.* 2006) and a negative regulatory element in intron 1 (Manohar *et al.* 1994). In addition, 3 miRNA binding site regions in the 3'-UTR (Ott *et al.* 2011) were analyzed in a similar way, but also here no nucleotide variants were found.

Table 5. Details of the PCR assays. PCR assays were performed as the above described RT-PCR assays, but only for 30 cycles. Sanger sequencing was performed as described above.

**Primer name**	**Primer sequence (5'->3')**	**Amplicon position (NC_009156.3)**	**PCR amplicon length (gDNA) Ta + PCR mix info**
ELN-12F<br>ELN-12R	GCAAGGCACTTGGGGATCCAGC<br>TCACTGCTGGCCCTGTTCCCG	11875615 (prom)<br>11876307 (prom)	693 bp<br>64°C
ELN-13F<br>ELN-13R	CTCCCTCCCGCTTCCTCTCCCG<br>TCCAGGCTGCGAGGGGTGGAC	11876227 (prom)<br>11876716 (exon 1, CDS)	490 bp<br>64°C
ELN-10F<br>ELN-1R	CCTACGAGGCAGCAATTACGCTTTGGG<br>TGCAACCTTGTAATCAACTTAACCCTGACC	11876564 (exon 1, 5'-UTR)<br>11877244 (intron 1)	681 bp<br>65°C + GC-rich
ELN-14F<br>ELN-14R	GGAAAGCCTGTGGCCGGAAGAGAA<br>GGGAGCAAGGGAGCCAAGAGATAGTGT	11907457 (exon 36, CDS)<br>11908013 (exon 36, 3'-UTR)	557 bp<br>65°C

7. Differential ELN mRNA expression analysis between case and control via RT-qPCR

Because amplicons from the control sample always appeared stronger on gel than from the case sample in the RT-PCR experiment, an ELN mRNA expression analysis between case and control was performed via RT-qPCR. An ELN primer pair targeting a part of the coding region from exon 1 (after the ATG) to exon 5 was used (Table 6). Averaged Cq values of the ELN

assay between the case and the control indeed showed that the control sample contained 10 times more ELN mRNA. However, when normalized, it was shown that both samples express similar ELN mRNA levels.

Table 6. Details of the RT-qPCR assay. The assay was performed as the above described qPCR assay for CNV analysis. The same RPL32 assay was used as reference assay.

**Primer name**	**Primer sequence (5'->3')**	**Amplicon position (XM_023655460.1)**	**RT-qPCR amplicon length (cDNA) Ta**
ELN-15F<br>ELN-10R	TGTCCATCGTCCACCCCTCGCA<br>GCCCGCCAACACCTGCCTTG	150 (exon 1, CDS)..<br>305 (exon 5)	156 bp<br>65°C

8. Identification of the 5'-end of the ELN mRNA in the case by adapter-free RACE (AF-RACE)

Because primers located in the 5'-UTR region of ELN could not amplify a RT-PCR product in the case, AF-RACE (based on Wang *et al.*, 2012; Figure 2) was performed to identify a potential alternative upstream exon in the case (Table 7). However, only fragments containing coding exon 1 sequences at their 5'-end could be retrieved. So, it appears that there is no upstream exon and that the ELN transcription start site in the case is positioned very close to its presumed start codon, in contrary to the control.

Figure 2. AF-RACE workflow (Wang *et al.*, 2012)

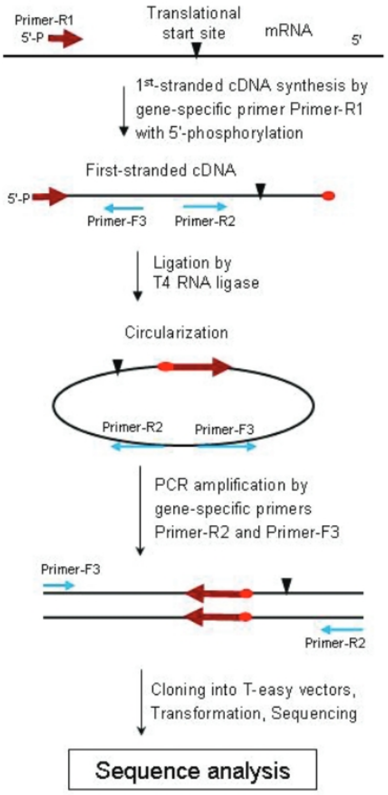

Table 7. Primers used in AF-RACE.

**Primer name**	**Primer sequence (5'->3')**	**Amplicon position (XM_023655460.1)**	**Amplicon length Ta**
ELN-15R (= Primer-R1 in scheme)	**P-**CAGCACCAGCCACTCCGCCAG	395 (exon 6)	na<br>68°C
ELN-16F (= Primer-F3 in scheme)<br>ELN-16R (= Primer-R2 in scheme)	CAGGGCTTGGGGCCTTTCCTG<br>GCCTTGGCTGGTTTGCCTCCA	327 (exon 5)<br>291 (exon 4)	variable (depending on 5'-end)<br>65°C

9. References

Altschul SF, Gish W, Miller W, Myers EW, Lipman DJ (1990) Basic local alignment search tool. *J Mol Biol*, **215**, 403-410.

Bashir MM, Indik Z, Yeh H, Ornstein-Goldstein N, Rosenbloom JC, Abrams W, Fazio M, Uitto J, Rosenbloom J (1989) Characterization of the complete human elastin gene. Delineation of unusual features in the 5'-flanking region. *J Biol Chem*, **264**, 8887-8891.

Bogaert L, Van Poucke M, De Baere C, Peelman L, Gasthuys F, Martens A (2006) Selection of a set of reliable reference genes for quantitative real-time PCR in normal equine skin and in equine sarcoids. *BMC Biotechnol*, **6**, 24.

Manohar A, Anwar RA (1994) Evidence for a cell-specific negative regulatory element in the first intron of the gene for bovine elastin. *Biochem J*. 1994, **300**, 147-152.

Okonechnikov K, Golosova O, Fursov M, the UGENE team (2012) Unipro UGENE: a unified bioinformatics toolkit. *Bioinformatics*, **28**, 1166-1167.

Ott CE, Grünhagen J, Jäger M, Horbelt D, Schwill S, Kallenbach K, Guo G, Manke T, Knaus P, Mundlos S, Robinson PN (2011) MicroRNAs differentially expressed in postnatal aortic development downregulate elastin via 3' UTR and coding-sequence binding sites. *PLoS One*, **6**, e16250.

Pierce RA, Moore CH, Arikan MC (2006) Positive transcriptional regulatory element located within exon 1 of elastin gene. *Am J Physiol Lung Cell Mol Physiol*, **291**, L391-399.

Van Poucke M, Peelman L (2017) Flexible, multi-use, PCR-based nucleic acid integrity assays based on the ubiquitin C gene. *bioRxiv*, http://dx.doi.org/10.1101/168195.

Wang C, Lee J, Deng Y, Tao F, Zhang LH (2012) ARF-TSS: an alternative method for identification of transcription start site in bacteria. *Biotechniques*, 52, doi: 10.2144/000113858.

Ye J, Coulouris G, Zaretskaya I, Cutcutache I, Rozen S, Madden T (2012) Primer-BLAST: A tool to design target-specific primers for polymerase chain reaction. *BMC Bioinformatics*, **13**, 134.

Figure 3. Schematic overview showing the equine ELN gene structure (NC_009156.3), its full-length mRNA (XM_023655460.1), amplified regions (CNV: copy number variation analysis; RE: analysis of regulatory elements in promoter, negative regulatory element in intron 1 and miRNA binding sites in the 3'-UTR; RT-PCR: analysis of transcript variants and nucleotide variants in the coding sequence; RT-qPCR: differential ELN mRNA expression analysis between case and control) and found nucleotide variants.

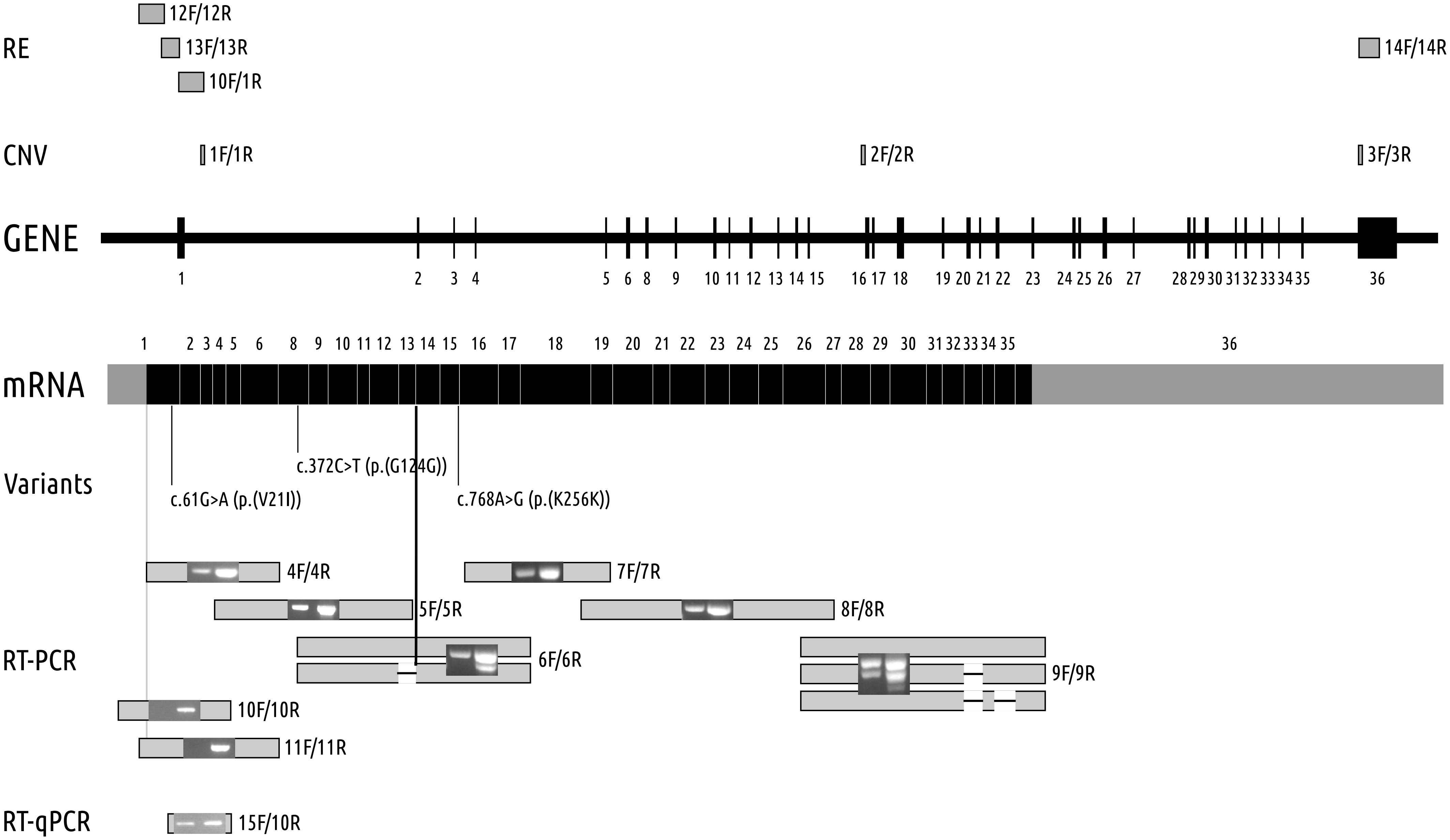

Supplement: Supplementary file 1 — AppendixS1 Supporting information [file JVIM-34-2152-s001.pdf]
